# Supplementary material for: Learning curve and surgical outcome of robotic assisted colorectal surgery with ERAS program
Source: Sci Rep. 2022 Nov 29;12:20566. doi: 10.1038/s41598-022-24665-w (PMC9709162; doi:10.1038/s41598-022-24665-w)
Supplement: Supplementary file 1 — Supplementary Information 1. [file 41598_2022_24665_MOESM1_ESM.zip › LSC Raw data-Ñ[▒K/142/O_ñw╝╨░O▒Kñσ.pdf]

正式摘要(電子簽章：000000228045840)

W83 / W83

臺中榮民總醫院

Operation Note

[redacted]

- 手術日期：2021/10/20
- 手術醫師：VS 林俊余，CR 陳璿夫，R 林順鴻，R 陳凱祥
- Anesthesia : GE
- Position : Lithotomy
- Aseptic agents : Soap , Betadine Aqua

Diagnosis

- Pre-operative diagnosis : Malignant tumor of Sigmoid colon, 30 CM FAV
  T1 lesion 20 cm FAV s/p EMR
- Post-operative diagnosis : Malignant tumor of Sigmoid colon, 30 CM FAV

  T1 lesion 20 cm FAV s/p EMR
  s/p laparoscopic AR.

Surgical Indication

- Removal of rectal tumor and pathological examination

Operation

- laparoscopic anterior resection for neogrowth of colorectum

Finding

- 1. a polypoid tumor 2*2 cm in size , sT2N0M0, T1 lesion 20 cm FAV s/p EMR
  2. LN(-) liver metastasis(-)
  3. margin: proximal 10 cm, distal 15cm.
  4. ascites(-) hemovac in pelvis.

Procedure

- Under GE, the patient was put in lithotomy position. 4 troca hole was made, the first was by open method. Pneumoperitonium was created. the Olympus Endo-EYE laparoscope was inserted from umbilical port. Abdominal exploration was done and the findings were mentioned above. The patient position was adjusted to head-down and right tilting to let small bowels move to RUQ and the pelvis was well exposured. The IMA was identified, dissected, skeletonized by triple clips and LigaSure energy device. The IMV was done in the same way. The mobilization of S-colon was done with dissection via embryonic plane between Gerota fascia and meso-sigmoid colon. The lateral attachement of sigmoid colon was also divided. After fully mobilization of S-colon, bilateral lateral ligment was divided by LigaSure. Transection of colon at R-S junction by 1 60 mm endocutters. The speciemn was removed from LLQ . The 29 mm envil was inserted in proximal colon annd take back into abdominal cavity. The end to end colorectal anastomosis was done by double stappling technique. After air-leak test, surgiwrap was left on LLQ. 5 ports and was closed in layers. Throughout the whole course of operation, patient stood quite well.
- Estimated blood loss: minimum
- 器械數量: 正確
- 紗布數量: 正確

Image

- PAINT
  - colon & rectum

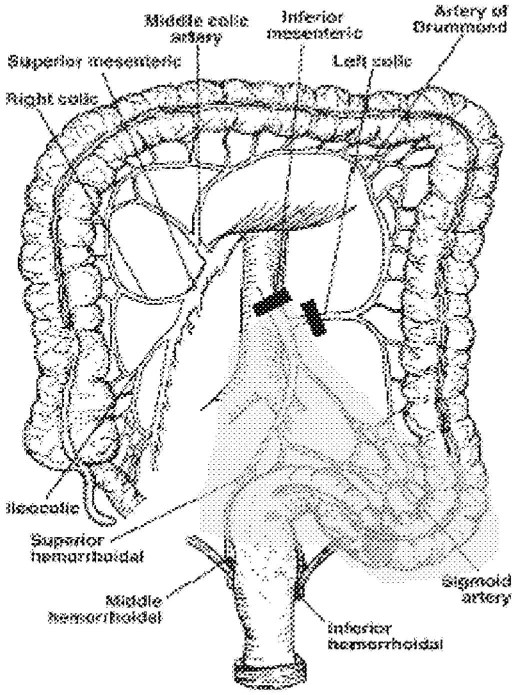

編輯主治 2222H林俊余

正式摘要(電子簽章：**000000228045840)**

W83 / W83

doctorRank:B
